# Influence of the Kinaesthetics care conception during patient handling on the development of musculoskeletal complaints and diseases – A scoping review

**DOI:** 10.1186/s12995-016-0113-x

**Published:** 2016-05-10

**Authors:** Alice Freiberg, Maria Girbig, Ulrike Euler, Julia Scharfe, Albert Nienhaus, Sonja Freitag, Andreas Seidler

**Affiliations:** Institute and Policlinic of Occupational and Social Medicine, Medical Faculty Carl Gustav Carus, Technische Universität Dresden, Fetscherstr. 74, Dresden, 01307 Germany; Department of Occupational Health Research, German Social Accident Insurance Institution for the Health and Welfare Service, Pappelallee 33-37, Hamburg, 22089 Germany; Institute for Health Service Research in Dermatology and Nursing, University Clinics Hamburg Eppendorf, Martinistr. 52, Hamburg, 20246 Germany

**Keywords:** Kinaesthetics, Musculoskeletal, Patient handling, Scoping review

## Abstract

**Abstract:**

The Kinaesthetics care conception is a nursing approach for patient handling which aims to prevent work-related complaints and diseases. The evidence about the influence of Kinaesthetics on musculoskeletal disorders among persons who handle patients is unclear to date. The purposes of the scoping review are to gain insight into the current state of research regarding the clinical effectiveness of Kinaesthetics (in terms of perceived exertion and musculoskeletal complaints) among persons who handle patients and to identify potential research gaps. A scoping review was conducted. The search strategy comprised a systematic search in electronic databases (MEDLINE, EMBASE, AMED, CINAHL), a hand search, a fast forward search (Web of Science) and a Google scholar-search. The review process was carried out independently by two reviewers. Methodological quality was assessed for all studies using three methodological main categories (reporting quality, internal validity, external validity). Thirteen studies with different study designs were included. Seven studies investigated musculoskeletal complaints and nine studies the perceived exertion of nursing staff. Most studies were of very low methodology. Most studies reported a decrease of musculoskeletal complaints and perceived exertion due to Kinaesthetics. In conclusion, there is only little evidence of very low quality about the effectiveness of Kinaesthetics. Out of the studies it could be assumed that Kinaesthetics may decrease the patient handling related perceived exertion and musculoskeletal pain of persons who handle patients. But an overestimation of these results is likely, due to inadequate methodology of included studies. As a result, no clear recommendations about the effectiveness of the Kinaesthetics care conception can be made yet. Since a research gap was shown, further high quality intervention studies are necessary for clarifying the effectiveness of Kinaesthetics.

**PROSPERO registry number:**

CRD42015015811

## Background

The Kinaesthetics care conception (in the following called “Kinaesthetics”) is an approach for patient handling which enables nursing staff to interact with patients in a way that shall protect themselves from injuries and that shall support their own as well as their patient’s health development [1]. Kinaesthetics is the study of the perception of human movements which are necessary for the execution of activities of daily life [1, 2]. The endeavor of Kinaesthetics is to divide all human action in its parts, which are called concepts: Interaction, functional anatomy, human movement, exertion, human functions and environment [1]. Patients shall be moved with spiral, not with parallel movements, because these require less effort [1]. In the theory of Kinaesthetics the human body consists of masses (bones) and spaces (muscles) [1]. If a handling person contacts the masses and moves them in a row, handling of a patient should be easier [1]. The theoretical framework of Kinaesthetics is based on the principles of behavioral cybernetics [1]. The concept was developed in the 1980s by Frank White Hatch and Linda Sue Maietta [2]. Training in Kinaesthetics is offered to nursing staff for about twenty years now [2]. It is applied in nursing [2], in infant handling (www.kinaesthetics.de), in palliative care [3], in education [2] and also in training of caregiving relatives [4]. There are several providers of Kinaesthetics which differ a lot in regard to quality, duration, qualification or curriculum of the training [5]. For example, in Germany the biggest and most known associations are the “European Kinaesthetics Association” and “MH Kinaesthetics Deutschland” [5].

Nursing staff has an increased risk for musculoskeletal disorders [6, 7]. A recently published review reported that musculoskeletal pain among nurses and nursing aids was highest in the lower back, followed by the shoulder joints and the neck (mean 12-month prevalence each: 55, 44, 42 %) [8]. Relative risk among nurses compared to clerks to suffer from low back pain is increased (point prevalence: 1.47 (95 % CI: 1.37–1.59)) [6]. Patient handling seems to be one of the risk factors for musculoskeletal disorders among nursing staff [9, 10]. One of the propagated effects of Kinaesthetics is the prevention of such physical complaints [2]. But the scientific evidence about the influence of Kinaesthetics on prevention of musculoskeletal complaints and diseases among persons who handle patients is unclear to date.

## Review

### Methods

#### Step 1—Identifying the research question

Since Kinaesthetics has not been studied much and it is a relatively new nursing intervention, a scoping review was conducted. The purposes of the scoping review are to gain insight into the current state of research regarding the clinical effectiveness of Kinaesthetics (in terms of perceived exertion and musculoskeletal complaints) among persons who handle patients and to identify possible research gaps. On the basis of these, the following research question arose:

“What is the scientific evidence about the influence of Kinaesthetics on the development of musculoskeletal complaints and diseases among persons who handle patients and is there a specific research gap?”

A general definition of scoping reviews does not exist [11, 12]. The purposes of scoping reviews are to map and to summarize the evidence of a certain research field and to identify research gaps [13]. On the basis of the results of a scoping review a systematic review can be conducted and recommendations for further research can be made [13]. In contrast to systematic reviews, scoping reviews are not restricted to certain study designs [13]. Furthermore, a critical appraisal of included studies is not intended [13], but its usefulness has been discussed in later methodology papers [12, 14]. Nevertheless, a transparent procedure is required to allow other researchers replication of study results [13, 15]. The results of a scoping review are summarized tabularly and descriptively [11].

This scoping review was conducted and structured based on methodological frameworks proposed by Arksey and O’Malley [13], and modified by Levac et al. [12] and Daudt et al. [14]. For checking if all relevant sections/topics of a review are reported, the PRISMA statement (Preferred Reporting Items for Systematic Reviews and Meta-Analyses) was used, since there is no specific reporting guideline for scoping reviews and most steps of this scoping review resemble the procedures of a systematic review [16]. The study protocol of this scoping review was published on the “International Register of systematic reviews” (PROSPERO) prior to study conduct (PROSPERO registry number: CRD42015015811) [17].

#### Step 2—Identifying relevant studies

A broad and sensitive search strategy was developed to identify as much relevant studies as possible. The following electronic databases were searched systematically:

- MEDLINE (via PubMed, from 1946 up to February 2nd 2016)
- EMBASE (via Ovid, from 1974 up to February 2nd 2016)
- AMED (via Ovid, from 1985 up to February 2nd 2016)
- CINAHL (via EBSCOhost, from 1982 up to February 2nd 2016)

The search strategy comprised terms for the population and the intervention. The individual terms of both categories were interconnected via the Boolean Operator “AND”. Search terms for the categories “outcome” and “study design” were not considered due to the aforementioned criteria of a scoping review [11]. The search string was first developed for MEDLINE and then adapted to the particular requirements of the other databases. Table 1 shows the search string for MEDLINE.

**Table 1:** Search string for MEDLINE

1	Nurses [All Fields] OR nurses[mh] OR nurse [All Fields] OR Allied Health Personnel [mh] OR Health Personnel [mh] OR physiotherapy* OR physical therap* OR therapist* OR occupational therap* OR family [mh] OR family [All Fields] OR relative [All Fields] OR “caregiving volunteer” [All Fields]
2	Kinaesthetics OR kinesthetics OR kinaesthetic OR kinesthetic OR kinesthesia OR kinaesthesia
3	#1 AND #2

Furthermore, a fast forward search was carried out via the Web of Science with the “Cited Reference Search”-function. For this purpose the references of all full texts that were included after title and abstract screening were used. In addition, a hand search for eligible studies was executed in the reference lists of full texts that were included after the title and abstract screening process and in the reference lists of topic related key articles and reviews.

In the later course of research it was decided to search Google scholar additionally, because it was assumed that further relevant studies could be retrieved. The search was conducted on September 27th 2015 using two terms (“Kinästhetik”, “kinaesthetics”) separately from each other. The first 500 hits each were screened by one reviewer (AF). If a reference seemed to be relevant, full text was retrieved and two reviewers (AF, MG/JS) decided about inclusion or exclusion.

The search results were organized with the electronic literature management program EndNote.

#### Step 3—Study selection

The PICOS-criteria (population, intervention, comparison, outcome, study design) were used to define the inclusion and exclusion criteria of this scoping review [16]. Healthcare workers as well as caregiving volunteers and family members who conduct patient handling activities on a regular basis, aged 15 to 70 years, working in all kinds of facilities where patient handling takes place, regardless of their qualification, were included. Kinaesthetics as individual measure or as part of a multimodal program was considered as intervention. No specification was made regarding the comparison. Relevant outcomes were all parameters that refer to musculoskeletal complaints and diseases, including the perceived exertion during or after patient handling. Initially it was planned to include patient parameters if they were also reported in the studies, but for better comprehensibility and structuring of the research project it was decided to exclude them. Since the scope of the review were clinical outcomes only, biomechanical studies were not considered. Editorials, commentaries, expert opinions and abstracts were excluded. No language restrictions were applied.

Title and abstract screening and full text screening were done independently by two reviewers (AF, UE/MG) in accordance with the inclusion and exclusion criteria. Disagreements were discussed and in case of a lack of agreement a third reviewer (AS) was consulted [12]. The title and abstract screening was tested in a pilot phase. All excluded studies of the full text screening were documented tabularly with reasons for exclusion. The proportion of observed agreement and Cohen’s Kappa were calculated to assess the agreement between the two reviewers [18].

#### Step 4—Charting the data

Data from included studies were extracted independently by two reviewers (AF, UE/MG) and discussed subsequently [11]. The process was piloted beforehand [12]. Relevant study information was documented in a standardized data extraction sheet. According to the iterative procedure of scoping reviews, the data extraction sheet was adapted to the identified data material in consultation with the reviewers.

#### Step 5—Collating, summarizing and reporting the data

##### Methodological quality assessment

As aforementioned, critical appraisal of included studies is still a subject of debate in methodology papers about scoping reviews [12–14]. To identify possible research gaps regarding the methodology of studies about Kinaesthetics and to give suggestions for methodological improvements for future research, it was decided to assess methodology of included studies in this scoping review. Due to the plethora of study designs methodological comparability of included studies seems hardly possible by using various appraisal tools for the appropriate study designs. According to the author’s knowledge one comprehensive checklist for several different study designs is missing. Thus, it was determined to evaluate three main categories (reporting quality, internal validity, external validity) for each study by two reviewers (AF, MG/JS) adjusted to each study type guided by categories/questions of the “Downs and Black checklist” [19] (for intervention studies) and the checklists of the “Critical Appraisal Skills Programmes“ (CASP) [20] (for reviews and qualitative studies), to obtain a methodological overview. Each main category is judged with “low risk of bias”, “high risk of bias” or “unclear risk of bias”. According to the Cochrane Handbook for Systematic Reviews of Interventions a bias is “a systematic error, or deviation from the truth, in results or inferences” [21]. “Low risk of bias” means that there is low risk of such a bias to occur in one of the main categories. “High risk of bias” means that the risk of such a bias is high. If information for judging a main category was reported insufficiently, the category was of “unclear risk of bias”.

Important key points for the assessment of the main category “Reporting quality” were sufficient information about study purpose, population, intervention, comparison, outcomes, results, etc. If these key points were not or poorly reported, this category was judged as “high risk of bias”.

According to the glossary of the German Network for Evidence-based Medicine (DNEbM) “Internal validity” refers to the extent to which the results of a study reflect the “true” effect of an intervention, i.e. are free of systematic bias and it is based on an optimal study planning, study conduct and study analysis [22]. For the main category “internal validity” study design specific key points (for intervention studies, qualitative studies or reviews) were given additionally. These key points were created after the designs of included studies were known (a posteriori).

The main category “External validity” is the degree to which the results of an observation withstand under other circumstances (e.g. population, setting) [23]. The basis for decision-making concerning the external validity of intervention studies were questions 11–13 of the “Downs and Black checklist” [19]. For the external validity of reviews question “8” of the CASP-review-checklist served as decision basis for judgement (“Can the results be applied to the local population?”) [20]. The following hints are given for answering the question: “The patients covered by the review could be sufficiently different to your population to cause concern.”; “Your local setting is likely to differ much from that of the review” [20]. Even though generalisation (and external validity) is considered important for qualitative research [24], this main category was not judged for qualitative studies, since appropriate reporting and appraisal checklists do not comprise suitable decision criteria [20, 25].

#### Data analysis

Retrieved data were analyzed for two main topics: study characteristics and study results. Study characteristics of interest were organizational data like year of publication, study language, place of study or setting; and research-related data concerning the PICOS-criteria [16]. Study results were distinguished between musculoskeletal complaints and perceived exertion of persons who handle patients. Data were summarized descriptively and tabularly.

#### Step 6—Consultation exercise

Since this step is optional, a consultation with experts and stakeholders was not undertaken.

## Results

### Study selection

The systematic database search yielded 1104 hits. After duplicate cleansing the titles and abstracts of 765 search results of the database search were screened. Of these, 736 studies were excluded and 29 studies were retrieved for screening the full texts. Twenty-five full texts were excluded due to an inadequate topic and/or article design (*n* = 18) [3–5, 26–38] or outcome (*n* = 7) [39–44]. Four studies were included in the scoping review [45–48]. Other search sources yielded additional nine hits. Via the fast forward search no further studies were identified. By checking the reference lists of included studies after full text screening and of topic related key articles six studies were found [49–54]. The Google scholar- search yielded three relevant studies [55–57]. Two studies of the hand search that seemed appropriate for inclusion according to their title and abstract were not deliverable via the Saxon State and University Library Dresden (SLUB) [58, 59]. For the title abstract screening of the database search an observed agreement of 0.98 and a Cohen’s kappa of 0.72 (Strength of agreement after Landis & Koch [60]: substantial) was calculated. The observed agreement for the full text screening process was 0.97 and the Cohen’s Kappa 0.87 (Strength of agreement after Landis & Koch [60]: almost perfect).

**Fig. 1:**
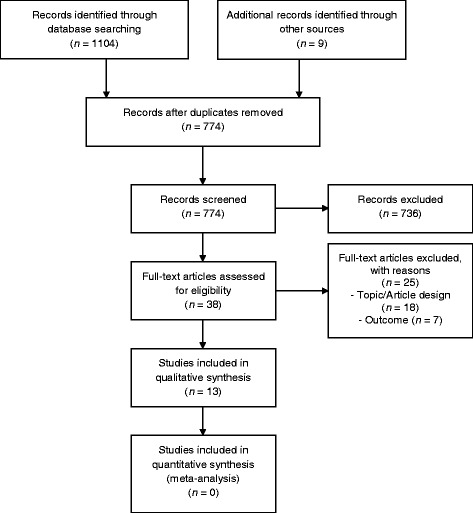
Flow diagram of the study selection

The results of the study selection are summarized with the PRISMA flow chart in Fig. 1. Please note that the number of hits for duplicate cleansing, title and abstract and full text screening contain additional records identified through other sources (in contrast to aforementioned numbers presented descriptively for database search only).

### Study characteristics

Thirteen studies were appropriate for the qualitative analysis: Two randomized controlled trials [47, 51], one cross-over study [48], one controlled before-after study [57], three uncontrolled before-after studies [46, 49, 52], three evaluation studies [45, 50, 53], one qualitative study [55] and two reviews [54, 56]. The difference between the before-after studies and the evaluation studies was that outcomes in the evaluation studies were only measured once (at the end of the project), whereas the outcomes of the before-after studies were measured at baseline and at follow-up. Data from the cross-over-study of Tamminen-Peter were only obtained from the first study part, to avoid a carry-over effect and hence the study can be considered as a non-randomized controlled trial [48]. Twelve studies were written in German and one in Finnish [48]. The author of the Finnish study, Leena Tamminen-Peter, provided additional documents for data extraction, which were translated by a professional translator. All studies were conducted in Europe, of these six in Germany, four in Austria, two in Switzerland and one in Finland. The study setting of nine studies was a hospital, one study took place in a retirement home and one in the homely environment of caregiving family members. One of the reviews included studies with hospitals and nursing homes as study setting [56], the other review did not define the setting of interest [54]. All studies were published after 2000, four of these after 2010. Nursing staff was the population of interest in all studies but one, which investigated caregiving family members [55]. Physical therapists were examined additionally in one study [50]. Only three studies provided patient characteristics [47, 48, 51]. Four studies examined a basic course of Kinaesthetics and three studies a comprehensive implementation of Kinaesthetics as intervention. None of these seven studies but one involved a comparison group. Another three studies investigated a specific patient handling task conducted with Kinaesthetics compared to the conventional handling method respectively the Durewall method (a nursing approach that uses jiu-jitsu principles). One study investigated the course “Kinaesthetics for caregiving family members”. The two reviews considered the concept “Kinaesthetics” in general without further confinements. For further information of study characteristics see Table 2.

**Table 2:** Summary of study characteristics

Study *(Language)*	Study Design	Setting, Place	Time frame/Duration	Population		Intervention	Comparison	Outcome of interest
				Persons who handle patients	Patients			
Betschon et al., 2014 *(German)* [45]	Evaluation Study	Nursing home, Meggen/Switzerland	Frame of the project: 2009–2012 Data collection: 2012	Nursing staff, Questionnaires *n* = 59 (Response: 75.0 %) Observations *n* = 17	NA	Basic course Kinaesthetics	NA	Physical Complaints, Perceived exertion immediately after mobilization^a^
Buge & Mahler, 2004 *(German)* [50]	Evaluation study	Nursing service, University Hospital, Heidelberg/Germany	Frame of the project: 2000–2003 Data collection: 2003	Nursing staff, *n* = 109; Physical therapists, *n* = 2 (Response: 33.7 %)	NA	Implementation of Kinaesthetics	NA	Feeling of physical relief (due to Kinaesthetics)^a^
Christen et al., 2002 *(German)* [46]	Uncontrolled before-after study	Hospital for nuclear medicine/radiotherapy, Zurich/Switzerland	Data collection: 1999 Follow-up: 6 month	Nursing staff, T0: *n* = 23 (Response: 92.0 %) T1: *n* = 20 (Response: 87.0 %) Data basis: *n* = 18	NA	Basic course Kinaesthetics	NA	Physical demands compared to subjective performance capacity^b^
Eisenschink et al., 2003 *(German)* [47]	Randomized controlled trial	Coronary care unit, University hospital, Ulm/Germany	Data collection: 1999–2000	Nursing staff, no further information	Patients after aortocoronary bypass surgery with sternotomy, I: *n* = 52 C: *n* = 50	Mobilisation of a patient with Kinaesthetics	Mobilisation of a patient with the standard mobilisation	Perceived exertion during first and second patient transfer^b^
Friess-Ott & Müller, 2006 *(German)* [53]	Evaluation study	University hospital, Heidelberg/Germany	Frame of the project: 1998–2003	Nursing staff, *n* = 159 (Response: 51.9 %)	NA	Basic course Kinaesthetics	NA	Pain, Physical relief, Effects on well-being^a^
Hock-Rummelhardt, 2013 *(German)* [57]	Controlled before-after study	Hospital, Vienna/ Austria	Frame of the project: 2010–2012 Follow-up: 20 month	Nursing staff, I: *n* = 15 C: *n* = 27 ^c^(Response: 17 %)	NA	Basic course Kinaesthetics, Practical guidance	No training in Kinaesthetics	Pain during/after nursing, Perceived exertion during work^a^
Huth et al., 2013 *(German)* [55]	Qualitative study (Interviews)	Homely environment, Witten/Germany	Data collection: 7 weeks	Caregiving family members, *n* = 10	NA	Course “Kinaes-thetics for caregiving family members”	NA	Musculo-skeletal complaints, Physical work load^a^
Lenker, 2008 *(German)* [51]	Randomized controlled trial	Intensive care unit, hospital, Ludwigsburg-Bietigheim/Germany	Data collection: 2002–2004	NM	Patients after abdominal laparotomy, I: *n* = 36 C: *n* = 38	Mobilisation of a patient to the edge of the bed based on Kinaesthetics principles	Mobilisation of a patient to the edge of the bed with conventional methods	Back pain during patient handling, Perceived exertion during patient handling^b^
Maietta & Resch-Kröll, 2009 *(German)* [49]	Uncontrolled before-after study	State hospital, Hörgas/Austria	Frame of the project: nearly 24 month	Nursing staff, T0: *n* = 92 T1: Response: 42.7 %	NA	Implementation of Kinaesthetics	NA	Perceived exertion during patient handling^a^
Rettenberger & Schoenemeier, 2005 *(German)* [52]	Uncontrolled before-after study	Hospital, Heidenheim/Germany	Frame of the project: 1999–2000 Follow-up: 14 month	Nursing staff, *n* = 43	NA	Implementation of Kinaesthetics	NA	Back complaints during daily patient handling, Sick leave due to low back or sciatic complaints^a^
Sedlak-Emperer, 2012 *(German)* [56]	Systematic review	Hospital, Nursing home, Austria	Search period: June 2009–March 2010 Applied publication period: 1990 – March 2010	Nursing staff from 18 years of age	Patients from 18 years of age	Kinaesthetics	Conventional nursing	Spinal complaints, Spinal loading^a^
Steinwidder & Lohrmann, 2008 *(German)* [54]	Narrative review	Setting: NM, Austria	Search period: July–September 2007 Applied publication period: NM	Nursing staff from 18 years of age	Patients from 18 years of age	Kinaesthetics	NM	Physical loading^b^
Tamminen-Peter, 2006 *(Finnish)* [48]	Non-randomized controlled trial	City hospital; I: Neurological rehabilitation C: Orthopaedic rehabilitation, Turku/Finland	Frame of the study: 2001–2002 Follow-up: 1 month	Nursing staff, *I* = 6 *C* = 6	Elderly, compliant, partially weight-bearing patients with little muscle strength and low ability to move, *n* = 18	Mobilisation of a patient from a wheelchair to bed with Kinaesthetics	Mobilisation of a patient from a wheelchair to bed with the Durewall method	Decrease of perceived strain of the lower back; Decrease of perceived strain of the shoulder joints^a^

*Abbreviations*: *C* control group, *I* intervention group, *n* number of participants, *NA* not applicable, *NM* not mentioned, *T0* start of the trial, *T1* end of the trial

^a^The outcome of interest was also a primary outcome in the study

^b^The outcome of interest was a secondary outcome in the study and only mentioned casually

^c^The paper contains different data about number of participants in the intervention and control group

### Methodological quality assessment

Details of methodological quality assessment of each study can be seen in Table 3.

**Table 3:** Methodological assessment of included studies

Study	Reporting quality	Internal validity	External validity
Betschon et al., 2014 [45]	HR	HR	HR
Buge & Mahler, 2004 [50]	LR	HR	LR
Christen et al., 2002 [46]	UR	HR	HR
Eisenschink et al., 2003 [47]	HR	UR	HR
Friess-Ott & Müller, 2006 [53]	HR	HR	HR
Hock-Rummelhardt, 2013 [57]	LR	HR	HR
Huth et al., 2013 [55]	LR	LR	NA
Lenker, 2008 [51]	HR	HR	HR
Maietta & Resch-Kröll, 2009 [49]	HR	HR	HR
Rettenberger & Schoenemeier, 2005 [52]	HR	HR	HR
Sedlak-Emperer, 2012 [56]	UR	HR	LR
Steinwidder & Lohrmann, 2008 [54]	HR	HR	UR
Tamminen-Peter, 2006 [48]	UR	HR	HR

General questions for each category

Reporting quality—Were important key points reported?

Internal validity—Are study results valid?

External validity—Are study results generalizable?

*Abbreviations*: *HR* high risk of bias, *LR* low risk of bias, *NA* not applicable, *UR* unclear risk of bias

The studies of Buge and Mahler and Huth et al. were the only two out of all included studies that were judged with “low risk of bias” in two categories each, Buge and Mahler for “Reporting quality” and “External validity” and Huth et al. for “Reporting quality” and “Internal validity” [50, 55]. Five studies received a “high risk of bias” in all three categories [45, 49, 51, 52, 53].

“Reporting quality” was at “low risk of bias” in three studies [50, 55, 57]. For three studies judgment of that category was unclear [46, 48, 56]. Although much important information in the systematic review of Sedlak-Emperer was reported (purpose, population, intervention, outcome, appraisal tools, study results), others were missing (study protocol, amount of reviewers, procedure of title-abstract- and full text screening) [56]. The same applies for the study of Christen et al [46]. Due to the Finnish language in the study of Tamminen-Peter (and a resulting language barrier), assessment of this category was “unclear” [48]. The remaining seven studies were evaluated with “high risk of bias” for “Reporting quality”, since too much important information (like population, setting, intervention, outcome measurements, statistical methods) was not or poorly reported. None of the studies did provide any information about a study protocol.

Only the qualitative study of Huth et al. was of “low risk of bias” in regard to “Internal validity” [55]. The category “Internal validity” was judged as “high risk of bias” for most of the studies (*n* = 11). In the evaluation studies a control group and a follow-up were missing [45, 50, 53]. As its name implies, a control group was missing in the uncontrolled before-after studies [46, 49, 52]. No randomization and no blinding were conducted in the non-randomized controlled trial of Tamminen-Peter and in the controlled before-after study of Hock-Rummelhardt [48, 57]. The randomized controlled trial of Lenker used an unconcealed allocation method [51]. In none of the two reviews a second reviewer was used [54, 56] and Steinwidder and Lohrmann did not appraise the methodology of included studies [54]. “Internal validity” of the randomized controlled trial of Eisenschink et al. is of “unclear risk of bias” due to missing or insufficient information regarding random sequence generation, allocation concealment and blinding of outcome assessment [47]. Most intervention studies recruited participants with a convenience sampling. Sample size was low in all included intervention studies (from *n* = 6 to *n* = 159). Most outcomes were obtained from subjective, self-reported data.

Two studies were evaluated as “low risk of bias” for the category “External validity” [50, 56]. The “External validity” of nine studies was judged as “high risk of bias”, because generalizability in terms of population and/or setting was questionable and as “unclear risk of bias” in one study due to insufficient information [54]. “External validity” in the qualitative study of Huth et al. was not judged [55].

Overall, the methodology of most included studies is of very low quality regarding reporting quality, internal validity and external validity.

### Study results

Detailed study results of the intervention studies are shown in Table 4. For each study, original (translated) terms are used. Details about measuring methods are shown in parenthesis. In the descriptive results part, results of intervention studies, the qualitative study and reviews are reported separately. Study results were not synthesized due to heterogeneity of included studies in many aspects (study design, outcome measures, intervention, etc.). Most results should be interpreted with caution due to very low methodological quality of included studies.

**Table 4:** Study results

Study (design, intervention)	Musculoskeletal complaints	Perceived exertion/physical loads
Betschon et al., 2014 [45]	Physical complaints: (% of surveyed nursing staff)	Perceived exertion immediately after mobilisation: (% of surveyed nursing staff)
(Evaluation study, Basic course Kinaesthetics)	- lower back/back: 39	- exhausting: 53
	- neck: 37	- very exhausting: 13
	- legs: 27	
Buge & Mahler, 2004 [50]	NA	Feeling of physical relief (due to Kinaesthetics)
(Evaluation study, Implementation of Kinaesthetics)		(Scale: 1–10, 1: Min; Measure: M, Mdn (SD))
		- cervical spine: 4.84, 5.00 (2.65)
		- arm/shoulder: 5.65, 6.00 (2.52)
		- elbow/wrist: 4.72, 5.00 (2.49)
		- thoracic spine: 6.00, 6.00 (2.42)
		- hip: 5.64, 6.00 (2.56)
		- knee: 5.26, 5.00 (2.73)
		- lumbar spine: 6.83, 8.00 (2.46)
Christen et al., 2002 [46]	NA	Physical demands compared to subjective capacity are…: (*N* = 18)
(Uncontrolled before-after study, Basic course Kinaesthetics)		…relatively tolerable:
		- never mentioned (T0, T1): *n* = 1
		- only mentioned at T0: *n* = 2
		- only mentioned at T1: *n* = 6
		- mentioned at T0 and T1: *n* = 9
		…(rather) too high:
		- never mentioned (T0, T1): *n* = 6
		- only mentioned at T0: *n* = 8
		- only mentioned at T1: *n* = 3
		- mentioned at T0 and T1: *n* = 1
Eisenschink et al., 2003 [47]	NA	Perceived exertion…: (Scale: 0–100, 100: not exhausting; Measure: Mdn)
(Randomized controlled trial, Mobilisation of a patient with Kinaesthetics)		…during first patient transfer:
		- I: 82.5
		- C: 37.0^a^ (*p* = 0.132)
		…during second patient transfer:
		- I: 84.5
		- C: 36.0^b^ (*p* = 0.0176)
Friess-Ott & Müller, 2006 [53]	Pain relief due to Kinaesthetics: (% of surveyed nursing staff)	NA
(Evaluation study, Basic course Kinaesthetics)	Full agreement:	
	- back: 38	
	- neck: 25	
	Partial agreement:	
	- neck, back, knee or legs: 23–36	
	No agreement:	
	- back: 16	
	- legs: 34	
Hock-Rummelhardt, 2013 [57]	Pain during/after nursing…: (Scale: 1–6, 1: no pain; Measure: M (SD))	Perceived exertion during work: (Scale: 1–6, 1: not exhausting; Measure: M (SD))
(Controlled before-after study, Basic course Kinaesthetics, practical guidance)	…at T0:	…at T0:
	- I: 2.36 (0.96)	- I: 4.07 (1.34)
	- C: 2.12 (1.04)^a^ (*p* = 0.615)	- C: 4.37 (1.25)^a,c^
	…at T1:	…at T1:
	- I: 2.05 (1.12)	- I: 4.27 (1.49)
	- C: 2.04 (0.90)^a^ (*p* = 0.974)	- C: 4.48 (1.48)^a^ (*p* = 0.505)
Lenker, 2008^d^ [51]	Back pain during patient handling (defined as pulling sensation): (*N* = 69)	Perceived exertion during patient handling: (*N* = 70)
(Randomized controlled trial, Mobilisation of a patient with Kinaesthetics)	- yes: I: *n* = 0; C: *n* = 9	- little: I: *n* = 33; C: *n* = 25
	- no: I: *n* = 33; C: *n* = 27^b,c^	- much: I: *n* = 0; C: *n* = 12^b,c^
Maietta & Resch-Kröll, 2009 [49]	NA	Perceived exertion during patient handling of…: (Scale: 1–6, 1: great effort; Measure: M)
(Uncontrolled before-after study, Implemen-tation of Kinaesthetics)		…care-dependent patients:
		- T0: 3.10
		- T1: 3.70 (Change: –19.4 %)^c^
		…obese patients:
		- T0: 2.05
		- T1: 3.15 (Change: –53.7 %)^c^
		…patients with high body tension:
		- T0: 2.28
		- T1: 2.91 (Change: –27.6 %)^c^
Rettenberger & Schoenemeier, 2005 [52]	Back complaints during daily patient handling: (% of surveyed nursing staff)	NA
(Uncontrolled before-after study, Implementation of Kinaesthetics)	- T0: 49	
	- T1: 30^c^	
Tamminen-Peter, 2006^d^ [48]	NA	Decrease of perceived exertion at T1 for…: (% of surveyed nursing staff)
(Non-randomized controlled trial, Mobilisation of a patient from wheelchair to bed with Kinaesthetics)		…lower back:
		- I: 71
		- C: 28^b^ (*p* < 0.01)
		…shoulder joints:
		- I: 53
		- C: 49^a,c^

*Abbreviation*: *C* control group, *I* intervention group, *M* mean, *Mdn* median, *Min* minimum, *N* total sample size, *n* sub-sample size, *NA* not applicable, *p* *p*-value, *SD* standard deviation, *T0* start of the trial, *T1* end of the trial

^a^No statistically significant difference between groups

^b^Statistically significant difference between groups

^c^No *p*-value provided

^d^Data were obtained from the author of the study

- Musculoskeletal complaints

Five intervention studies, one qualitative study and one review asked for musculoskeletal complaints of the nursing staff [45, 51–53, 55–57].

### Musculoskeletal complaints—Intervention studies

The randomized controlled trial of Lenker reported a statistically significant difference (no *p*-value provided) for a more frequent occurrence of back pain during patient handling in the control group in comparison to the intervention group [51]. Hock-Rummelhardt found no statistically significant difference, neither between the intervention group and the control group at follow-up (*p =* 0.974), nor in the intervention group over time (*p =* 0.308) for pain during/after nursing [57]. In the study of Rettenberger and Schoenemeier back complaints during patient handling decreased from 49 to 30 % over time (no absolute numbers and *p*-value provided) [52]. Furthermore, sick leave due to low back or sciatic pain declined from start to end of the trial. Of those with back complaints during patient handling, 44 % took sick leave at the start of the trial, but only 4.4 % at follow-up. Betschon et al. reported that nursing staff that participated in a basic course of Kinaesthetics mainly felt physical complaints of the lower back/back (39 %), the neck (37 %) and the legs (27 %) immediately after patient handling (no absolute numbers provided) [45]. It is unclear, whether these numerical data are a sign for symptom improvement or for adverse effects of Kinaesthetics since no comparative values (before-after or of a control group) were reported. In the study of Friess-Ott and Müller, after attending a basic course of Kinaesthetics, 38 % respectively 25 % of the nursing staff fully agreed, that they had less pain than before the course in the back respectively the neck; 23 % to 36 % felt partial pain relief of the neck, back, knees or legs; and 16 % respectively 34 % felt no pain relief in the back respectively the legs (no absolute numbers provided) [53]. In summary, most intervention studies reported an improvement of musculoskeletal complaints (between groups and/or over time) in nursing staff due to Kinaesthetics. No adverse effects were reported.

### Musculoskeletal complaints—Qualitative study

Participants in the qualitative study of Huth et al. reported a reduction of pain and muscular tension and acknowledged the preventive character of Kinaesthetics (related to musculoskeletal complaints) [55].

### Musculoskeletal complaints—Review

Regarding musculoskeletal complaints the systematic review of Sedlak-Emperer reported about two studies that emphasized the preventive and rehabilitative character of Kinaesthetics (concerning spinal complaints) [52, 56].

b) Perceived exertion/physical loads

Eight intervention studies, one qualitative study and two reviews described the perceived exertion or physical loads of the nursing staff [45–51, 54–57].

### Perceived exertion/physical loads—Intervention studies

In the randomized controlled trial of Eisenschink et al. the perceived exertion during a specific patient handling task with Kinaesthetics after an aortocoronary bypass surgery was rated lower than handling with the standard mobilisation [47]. During second patient transfer this difference was statistically significant (*p =* 0.0176), but not during first patient transfer (*p* = 0.132). It should be noted critically that the intervention group comprised more patients with movement restrictions than the control group (37 % versus 15 %). Similar results were seen in the randomized controlled trial of Lenker [51]. Hock-Rummelhardt observed no statistically significant difference of the perceived exertion during work between groups at follow-up (*p =* 0.505) or in the intervention group from start to end of the trial (*p =* 0.490) due to Kinaesthetics [57]. For perceived exertion of the lower spine during a specific patient handling task a statistically significant higher reduction was reported with application of Kinaesthetics in comparison with the Durewall method in the non-randomized control trial of Tamminen-Peter (*p* < 0.01) (no absolute numbers provided) [48]. Such a difference between the intervention and the control group was not seen for the reduction of perceived exertion of the shoulder joints. In a before-after study of Christen et al. physical demands of work were described mainly as too high at baseline and as relatively tolerable especially at follow-up [46]. In another before-after study, Maietta and Resch-Kröll reported the reduction of the perceived exertion during patient handling for different kinds of patients (care-dependent patients, obese patients, patients with high body tension) from baseline to follow-up (no *p*-values provided) [49]. Betschon et al. reported that 53 % of respondents felt exhausted immediately after mobilisation and 13 % felt very exhausted after attending a basic course of Kinaesthetics (no absolute numbers provided) [45]. As aforementioned no comparative values were available, so that an interpretation of these results is difficult. After an implementation of Kinaesthetics, 52.8 % of the participants stated a high degree of physical relief for the lumbar spine (a value of 8 to 10 on a 10-point-scale with ”1” meaning no “physical relief”) in the evaluation study of Buge and Mahler [50]. Overall, in all but two intervention studies a reduction of perceived exertion due to Kinaesthetics was observed [45, 57].

### Perceived exertion/physical loads—Qualitative study

Participating family members in the qualitative study of Huth et al. noticed a reduction of physical work load due to Kinaesthetics [55]. They also mentioned that due to handling of a family member with Kinaesthetics, lifting and carrying can be avoided.

### Perceived exertion/physical loads—Reviews

The systematic review of Sedlak-Emperer included six studies that suggest the spine-gentle aspects of Kinasthetics [56] and the included studies in the review of Steinwidder and Lohrmann showed a lowered physical load due to Kinaesthetics (especially of the spine) [54].

## Discussion

To date, only little evidence about the influence of Kinaesthetics of very low quality exists. Based on the results of included studies, it might be assumed that Kinaesthetics could reduce the perceived exertion during patient handling especially for the lower back and could decrease musculoskeletal pain in general and during patient handling activities in persons who handle patients. An overestimation of the results is likely due to the inadequate methodology of studies. A selection bias is existent in most intervention studies, since convenience sampling occurred. Possibly more participants that had a positive attitude towards Kinaesthetics attended. Further, the power of all included intervention studies is questionable due to low sample sizes.

The systematic review of Sedlak-Emperer comprised seven of the ten intervention studies that were included in this scoping review [46–52]. Most of these studies dealt with the outcome of perceived exertion of nursing staff and were also included in this review, but only two studies dealt with musculoskeletal complaints of nursing staff [52, 58], of which one could not be retrieved for this scoping review [58]. Results of this systematic review are in line with the results of this scoping review concerning the decrease of perceived exertion and the musculoskeletal pain of nursing personnel due to Kinaesthetics [56]. Of the included studies in the narrative review of Steinwidder and Lohrmann [54] only one study met the inclusion criteria of this scoping review and hence was included [48].

Kinaesthetics shall also impact patients, not only persons who handle patients. But this was not the focus of this scoping review. Some of the included studies also evaluated parameters of patients [47, 49–52], but reported only few effects due to Kinaesthetics.

Concerning the methodological quality of included studies, most studies were judged as “high risk of bias” in regard to “Reporting quality”, “Internal validity” and “External validity”. “Reporting quality” was insufficient in seven studies, because important study information such as details about the population, intervention or outcome measures was not provided. Most studies had bias (or systematic error) in regard to study conduct and study analysis, thus were of “high risk of bias” for “Internal validity”. Important methodological aspects were not fulfilled in the intervention studies (e.g. randomisation, concealed allocation, blinding) and reviews (e.g. use of a second reviewer). Most of the results of included studies seem not to endure under other circumstances (e.g. population, setting) than applied in the individual studies and were therefore rated as “high risk of bias” for “External validity”. Even though results of the qualitative study of Huth et al. could eventually be transferred to care situations of other caregiving family members, it was decided not to assess “External validity” of this study design due to aforementioned reasons.

Only six of the 13 identified studies were indexed in the searched electronic databases (of which four were found with the electronic literature search). Further nine studies were found by hand search (*n* = 6) and via Google scholar (*n* = 3).

Only studies from Europe, mainly from German speaking countries, were included. It seems that the use of this nursing intervention is distributed primarily in these countries, since literature and training courses about Kinaesthetics are widely spread in Germany [32] and the European Kinaesthetics association comprises amongst few others the country organizations of Germany, Switzerland and Austria [2].

The comparability between the included studies is questionable, since different kinds of interventions (basic course of Kinaesthetics, implementation of Kinaesthetics, and execution of specific patient handling tasks with Kinaesthetics) different study designs, different types of patients and different outcome measures were applied.

It should be noted critically that a standardization of such an individual nursing method like Kinaesthetics is very difficult to ensure [61]. Further, the concept is a complex intervention [62], and not just a simple transfer and lifting technique [5].

One influencing factor of the effectiveness of Kinaesthetics in daily practice is its challenging implementation into the clinical setting [40]. Thus, various supporting respectively inhibiting factors should be taken into account, such as a good team that is willing and motivated to implement the concept, the conduct of case discussions, workshops and practical guidance or evident success respectively lack of time, rejection of the concept or fear of innovation [5, 40, 62, 63]. Training of Kinaesthetics is furthermore of little benefit if it isn’t integrated into the organizational framework of a healthcare facility [5, 28], since focusing exclusively on knowledge transfer does not meet the complexity of the implementation process [63].

Based on the findings of this scoping review, the conduct of a subsequent systematic review for our research question is not indicated, since finding further relevant studies is not expected (due to the excessive search strategy of this scoping review).

One resulting research gap is the lack of high-quality research about the clinical effectiveness of Kinaesthetics in preventing musculoskeletal disorders among persons who handle patients. Thus, high-quality intervention studies, in form of cluster-randomized trials or randomized controlled trials in different settings with different health care workers, are needed to fill this research gap.

### Strengths and weaknesses of the review

This is the first comprehensive overview of evidence (conducted as a scoping review) about the influence of Kinaesthetics on persons who handle patients with the same systematic and rigorous methodology used in systematic reviews that used two independent reviewers during the whole review procedure and included qualitative studies as well as reviews, in contrast to the reviews of Sedlak-Emperer [56] or Steinwidder and Lohrmann [54].

The extensive and sensitive search strategy using various sources was useful in identifying many grey literature studies about the influence of Kinaesthetics on persons who handle patients (especially hand search and Google scholar search).

Despite the heterogeneity of study designs, three main categories of methodology (reporting quality, internal validity, external validity) of each study (design) were evaluated independently by two reviewers, to ensure comparability of methodological quality of all included studies. Since this approach was utilized for the first time, no validity and reliability values are available. Even though, critical appraisal of included studies in scoping reviews was initially not intended [12, 13], later methodology papers recommend it [14, 64]. But, none of these methodology papers addressed the problem of appraisal and simultaneous comparison of different kinds of study designs.

Synthesis of study results was not possible due to heterogeneity of included studies. In general, study results of included studies are not synthesized, but summarized descriptively in scoping reviews [12–14].

## Conclusions

The propagated positive effects of Kinaesthetics can only be assumed according to the findings of this scoping review. Kinaesthetics seems to decrease the perceived exertion and musculoskeletal pain of persons who handle patients. But since most included studies are of poor methodological quality an overestimation of these effects is likely. As a result, no clear recommendations about the effectiveness of Kinaesthetics on persons who handle patients can be made yet. Since a research gap was shown for the effectiveness of Kinaesthetics on persons who handle patients, further high quality intervention studies are necessary for clarifying this issue.

## Abbreviations

AF: Alice Freiberg
AN: Albert Nienhaus
AS: Andreas Seidler
AMED: The Allied and Complementary Medicine Database
CASP: Critical Appraisal Skills Programmes
CINAHL: Cumulative Index to Nursing and Allied Health Literature
DNEbM: German Network for Evidence-based Medicine
e.g.: exempli gratia
EMBASE: Excerpta Medica Database
et al.: et alii
etc.: et cetera
i.e.: id est
JS: Julia Scharfe
MEDLINE: Medical Literature Analysis and Retrieval System Online
MG: Maria Girbig
n: number
p: p-value
PICOS: population, intervention, comparison, outcome, study design
PRISMA: Preferred Reporting Items for Systematic Reviews and Meta-Analyses
PROSPERO: International Register of systematic reviews
SF: Sonja Freitag
SLUB: Saxon State and University Library Dresden
UE: Ulrike Euler
95%CI: 95 % confidence interval
